# Significant association between joint ultrasonographic parameters and synovial inflammatory factors in rheumatoid arthritis

**DOI:** 10.1186/s13075-018-1802-x

**Published:** 2019-01-10

**Authors:** Yasushi Kondo, Katsuya Suzuki, Yumiko Inoue, Koumei Sakata, Chihiro Takahashi, Masaru Takeshita, Yoshiaki Kassai, Takahiro Miyazaki, Rimpei Morita, Yasuo Niki, Yuko Kaneko, Hidekata Yasuoka, Kunihiro Yamaoka, Akihiko Yoshimura, Tsutomu Takeuchi

**Affiliations:** 1 Division of Rheumatology, Department of Internal Medicine, Keio University School of Medicine, 35 Shinanomachi, Shinjuku-ku, Tokyo, Japan; 2 Immunology Unit, Research, Takeda Pharmaceutical Company Limited, Fujisawa, Kanagawa, Japan; 3 Department of Microbiology and Immunology, Keio University School of Medicine, Tokyo, Japan; 4 Department of Orthopedic Surgery, Keio University School of Medicine, Tokyo, Japan; 5 Nektar Therapeutics, San Francisco, CA USA

**Keywords:** Ultrasonography, Rheumatoid arthritis/DG, Cytokines, Inflammation, Diagnostic imaging

## Abstract

**Background:**

Ultrasonography (US) can directly demonstrate joint inflammation, including grayscale (GS) signs of synovial hypertrophy and power Doppler (PD) techniques to demonstrate increased blood flow and vascularization. Recently, echogenicity, especially hypoechoic synovium, has also been associated with local inflammatory activity. However, only a few studies have demonstrated correlation between histopathologic and immunopathologic evaluation and US findings. The aim of this study was to clarify whether joint US findings including synovial hypertrophy, vascularity, and echogenicity can accurately characterize synovial pathophysiology in patients with active rheumatoid arthritis (RA).

**Methods:**

A total of 44 patients with RA were included, both treated (*n* = 25) and untreated (*n* = 19) and scheduled for US examination of the knee joint with synovial fluid (SF) aspiration and two treated patients also underwent synovial biopsy. US images were quantitatively analyzed using grayscale assessment of synovial hypertrophy and PD for vascularity and echogenicity. Levels of nine SF cytokines and growth factors were also measured.

**Results:**

Both US synovial hypertrophy and PD vascularity significantly correlated with SF inflammatory cytokine levels such as IL-6, IL-8, IL-1β and IL-10 in untreated patients. Angiogenic factors, including vascular endothelial growth factor (VEGF), only correlated with PD vascularity. In the treated patients, the associations between synovial hypertrophy and any cytokines were diminished, although synovial vascularity and echogenicity correlated with IL-6 and VEGF (*p* < 0.05). Histopathologic analysis revealed that hypoechogenicity of the synovium correlated with marked infiltration of lymphocytes and hypervascularity.

**Conclusions:**

We demonstrated the pathophysiological origins of US findings in the joint. The degree of US vascularity of the synovium correlated with local inflammatory cytokine levels and angiogenetic factors in patients with active RA. Synovial echogenicity, and not hypertrophy, correlated with inflammation, especially in treated patients with RA.

**Electronic supplementary material:**

The online version of this article (10.1186/s13075-018-1802-x) contains supplementary material, which is available to authorized users.

## Introduction

Ultrasonography (US) can directly demonstrate joint inflammation, including synovial hypertrophy, using grayscale (GS), and increased blood flow and vascularization demonstrated using power Doppler (PD) techniques [1–4]. PD findings are held to be more reflective of joint inflammation than GS in the clinical setting, and the persistence of synovitis with PD activity suggests disease flare and progression of joint damage [5, 6]. Additionally, abnormal GS findings can also represent the residue of past synovial inflammation, and it has been suggested that the echogenicity, especially hypoechoic synovium, correlates with local inflammatory activity [7, 8]. However, only a few studies have demonstrated correlation between histopathologic and immunopathologic evaluation and US findings [9, 10]. More recent studies showed that PD signals are closely associated with synovial vascularity although they have not consistently been associated with histologic findings of inflammation, with some discordant results [11–16]. One of the reasons for the above is sampling bias and the heterogeneity inherent in synovial tissue. Most of the synovial tissue samples in these previous studies were obtained from knee arthroplasty in patients with long disease duration and/or modification by various medications [17, 18]. Therefore, the question of which US findings including synovial hypertrophy, vascularity and echogenicity more accurately reflect local pathophysiology in RA joints remains uncertain [19].

Synovial fluid (SF) is easily accessible. It contains inflammatory proteins that may provide more specific information on the affected joint than those from peripheral blood [20–23]. SF cytokines and growth factors produced by inflamed synovial tissues and infiltrating cells play an important role in the local pathogenesis of RA. These are more abundant and form different networks from those in plasma [24]. Based on this, SF samples may be more helpful than peripheral blood in assessing joint inflammation in a clinical setting, provided the association between SF contents and joint US findings can be clarified.

In this study, to clarify the accuracy of US in synovial hypertrophy, vascularity, and echogenicity in active RA, we measured SF cytokines and growth factors and looked for associations with corresponding US findings in patients with active RA.

## Methods

### Patients

Forty-four consecutive patients with RA were recruited from Keio University Hospital. All patients fulfilled the American College of Rheumatology (ACR) 1987 revised criteria for the classification of RA or the 2010 ACR/European League Against (EULAR) RA classification criteria with clinically active synovitis in the knee joint [25, 26]. Demographic and clinical characteristics were obtained from medical records, including age, sex, arthritic symptom duration, tender joint count, swollen joint count, erythrocyte sedimentation rate (ESR), C-reactive protein (CRP), matrix metalloprotease (MMP)-3, rheumatoid factor (RF), anti-cyclic citrullinated peptide antibody (anti-CCP), and mean disease activity score using the 28-joint disease activity score/erythrocyte sedimentation rate (DAS28-ESR).

We divided the patients into untreated and treated groups. Untreated patients were defined as having never been treated with any type of disease-modifying anti-rheumatic drugs (DMARDs) or with corticosteroids. Treated patients with RA were defined as receiving any DMARD at the time of US examination and SF aspiration.

All patients were scheduled to undergo knee US examination and US-guided arthrocentesis on the same day. Two treated patients who had SF aspiration also underwent US-guided synovial biopsy from the supra-patellar pouch. This study was approved by the ethics committee of Keio University and written informed consent was obtained from all patients.

### Ultrasound examination

US scans of the knee joint were performed, and the images were recorded by a single experienced operator (YK) using the Noblus (Hitachi Medical Corporation, Tokyo, Japan) with a L64 linear type probe (5–18 MHz), with the high definition dynamic tissue harmonic imaging - penetration (HdTHI-P) setting. Patients’ knees were positioned with 30 degrees of flexion during US procedure. Three standardized sites were assessed in the knee joint, including the lateral supra-patellar pouch, lateral recess, and medial recess, and representative one US image for each site that demonstrated the maximum synovial hypertrophy by GS and vascularity by PD was stored (three images per subject in total were stored). The gain, time gain compensation, and depth were preserved at the default settings during the study period to allow later quantitative analysis. For PD settings, pulse repetition frequency was adjusted to the lowest value for the anatomic area scanned and color gain was set just below the level at which color noise appeared. US images were scored by two independent rheumatologists (YI and YK), who are experienced in US analysis, while blinded to patient clinical information.

### Ultrasound image scoring and quantitative analysis

Semi-quantitative scoring of synovial hypertrophy (GS score) and vascularity (PD score) was applied using a scale of 0 (none) to 3 (severe) [14, 18–27]. The intraclass correlation coefficients (ICC) of the semi-quantitative GS score and PD score were in good agreement (GS score 0.82 and PD score 0.90) [28].

Quantitative analysis of the recorded US images was performed using the Image J open source image processing program designed for scientific multidimensional images (Image processing and analysis in Java, https://imagej.nih.gov/ij/). This software facilitates manual drawing of outlines of the synovial tissue and blood flow in the US images. It also gives the results of quantitative measurement of pixel counts and gray values (brightness) within the selected regions of interest (ROIs). Hypertrophy was defined as pixel counts (area) of US synovial hypertrophic tissue, carefully excluding synovial fluid. Vascularity was defined as area of power Doppler signal [14]. Echogenicity, defined as the mean of 8-bit brightness values of 0–255 within the synovial hypertrophic area, was also measured. To represent absolute US synovial echogenicity objectively, we converted the mean brightness to calibrated optical density according to the tutorial in the Image J user guide (https://imagej.nih.gov/ij/docs/user-guide.pdf). The mean of the hypertrophy, echogenicity, and vascularity values was calculated in the three sites of each knee joint.

### Measurement of synovial fluid cytokines and growth factors

SF samples (1 mL) were immediately treated with 1 μg/mL hyaluronidase for 30 min after arthrocentesis and centrifuged (300 *g*/5 min). The supernatant was centrifuged (3000 *g*/10 min) to remove insoluble substances. Samples were stored at − 80 °C until analysis. SF samples that contained visible blood were carefully excluded. Concentrations of SF cytokines including growth factors, interleukin (IL)-6, vascular endothelial growth factor (VEGF), IL-8, tumor necrosis factor (TNF)-α, IL-1β, IL-10, IL-17A, fractalkine, and granzyme B were measured with a cytometric bead array (BD Biosciences, San Jose CA, USA).

### Ultrasound-guided synovial biopsy and histopathology

US-guided knee synovial biopsies were performed as scheduled using the same US equipment for guidance. Each biopsy was performed according to the technique described by Kelly et al., [14] using a 16G semi-automatic biopsy system (TSK Laboratory, Tochigi, Japan). Tissue was harvested from the lateral aspect of the supra-patellar pouch [29]. Each synovial specimen was divided into two parts, one for gross examination and the second for histologic preparation and staining with hematoxylin and eosin (H&E).

### Statistical analysis

All statistical analyses were performed using JMP 11 (SAS Institute Inc., Cary NC, USA). Continuous data are presented as the mean with standard deviation (SD) or median with interquartile range (IQR). Data were compared using Fisher’s exact test or the Wilcoxon signed-rank test, as appropriate. Correlation was tested using Spearman’s method. Multivariate linear regression analysis was conducted with up to three variables with *p* values <0.05 in univariate analysis, including IL-6, VEGF, and IL-10 as independent variables, with a variance inflation factor of < 5 for validation of multicollinearity in linear regression models. The Kruskal-Wallis test was applied for analysis of variance.

## Results

### Patient characteristics and validation of US quantitative measurement

Patient demographics, ultrasonography data, and synovial fluid cytokines for 44 patients are shown in Additional file 1: Table S1. There were 37 female patients (84%); mean age was 64 years, and median symptom duration was 1.5 years. Mean DAS28-ESR was 5.2, indicating high disease activity. There were 33 (75%) and 34 (77%) who were positive for serum RF and anti-citrullinated protein antibodies (ACPA), respectively. First, we analyzed the correlation between quantitative data and corresponding US scoring to validate the US findings (Additional file 2: Figure S1). Both synovial hypertrophy and vascularity, which were analyzed by image J software, were closely correlated with standard US scoring. (hypertrophy and GS score; rho = 0.79, vascularity and PD score; rho = 0.89), Therefore, we confirmed that those quantitative US parameters were appropriate to use in the following analysis.

### Association between US findings and synovial fluid cytokine and growth factors in patients with RA

We conducted separate correlation analysis of the untreated and treated RA patient groups. Characteristics of both untreated and treated RA groups are shown in Table 1. Nineteen patients were classified as untreated and 25 as treated patients with RA. Patients in the treated group had been administered DMARDs, including methotrexate (92%), corticosteroids (24%), and anti-TNF agents (42%). Overall clinical characteristics were well-matched in the two groups, except for disease duration and SF cytokine concentration.

**Table 1:** Patient characteristics, ultrasonography, and synovial fluid cytokines

Characteristics	Untreated (*n* = 19)	Treated (*n* = 25)	*p* value
Demographics			
Age (years)	59 (49–71)	64 (53–73)	n.s
Female *n* (%)	14 (73%)	23 (92%)	–
BMI	22.8 (18.1–26.3)	23.1 (17.5–29.4)	
RF positivity *n* (%)	13 (68%)	20 (80%)	–
ACPA positivity *n* (%)	12 (63%)	22 (88%)	–
DMARDs *n* (%)	0 (0%)	23 (92%)	–
Corticosteroids *n* (%)	0 (0%)	6 (24%)	–
Anti-TNF biologics *n* (%)	0 (0%)	10 (40%)	–
Disease duration (years)	0.3 (0.25–2)	5.0 (0.7–10.5)	0.004
DAS28	5.6 ± 1.2	5.3 ± 1.4	n.s
CRP (mg/dL)	4.0 ± 2.9	3.8 ± 2.6	n.s
ESR (mm/h)	88.2 ± 35.2	83.7 ± 35.0	n.s
MMP3 (ng/mL)	368.3 ± 305.6	370.4 ± 288.1	n.s
Synovial fluid cell count/μL	6561 ± 6873.8	7138 ± 4503.3	n.s
Ultrasonography findings			
GSUS score	2.3 (2.0–2.7)	2.3 (2.2–2.8)	n.s
PDUS score	2.0 (1.7–2.7)	1.9 (1.5–2.0)	n.s
Hypertrophy (×10^3^ pixel)	141.3 ± 49.5	135.2 ± 43.3	n.s
Vascularity (×10^3^ pixel)	21.6 ± 16.3	16.8 ± 14.2	n.s
Echogenicity	40.3 ± 4.7	51.4 ± 5.7	0.009
Synovial fluid cytokines			
IL-6 (ng/mL)	25.1 ± 25.7	23.2 ± 24.3	n.s
VEGF (pg/mL)	611.3 ± 530.1	569.9 ± 522.3	n.s
TNF-α (pg/mL)	151.8 ± 449.9	132.6 ± 389.1	n.s
IL-8 (pg/mL)	1363.8 ± 1403.9	1999.1 ± 2045.3	n.s
IL-1β (pg/mL)	26.1 ± 29.6	41.4 ± 36.2	n.s
IL-10 (pg/mL)	29.8 ± 22.0	33.7 ± 18.9	n.s
IL-17A (pg/mL)	36.2 ± 61.6	49.9 ± 63.5	n.s
Granzyme B (pg/mL)	74.7 ± 93.9	89.7 ± 89.2	n.s
Fractalkine (pg/mL)	78.9 ± 75.3	83.9 ± 83.8	n.s

Values are mean ± SD or median (IQR) unless otherwise specified

*BMI* body mass index, *RF* rheumatoid factor, *ACPA* anti-citrullinated protein antibody, *DMARDs* disease-modifying anti-rheumatic drugs, *DAS28* disease activity score based on 28 joints, *CRP* C-reactive protein, *ESR* erythrocyte sedimentation rate, *MMP-3* matrix metalloproteinase-3, *GSUS* grayscale ultrasound score, *PDUS* power Doppler ultrasound score, *hypertrophy* quantitative synovial grayscale area (×10^3^ pixels), *vascularity* quantitative synovial power Doppler area (× 10^3^ pixels), *echogenicity* quantitative gray value of synovial area, *IL* interleukin, *VEGF* vascular endothelial growth factor, *TNF* tumor necrosis factor, *n.s.* not significant

Univariate correlation analysis in untreated patients with RA showed that US findings of synovial hypertrophy and vascularity were positively associated with several SF cytokines and growth factors, including IL-6, IL-1β, IL-10, IL-17A and Granzyme B, as shown in Table 2. VEGF and the vascular chemokine fractalkine correlated specifically with synovial vascularity. Synovial echogenicity was inversely correlated with SF VEGF (*p* < 0.05).

**Table 2:** Correlation between ultrasonography and synovial fluid cytokines in patients with RA

	Untreated (*n* = 19)			Treated (*n* = 25)		
	Hypertrophy	Vascularity	Echogenicity	Hypertrophy	Vascularity	Echogenicity
IL-6	0.75^*^	0.80^*^	0.01	0.38	0.65^*^	−0.46^*^
VEGF	0.21	0.68^*^	0.15	0.28	0.43^*^	−0.55^*^
IL-8	0.30	0.41	− 0.15	0.13	0.33	−0.17
TNF-α	0.29	0.37	0.01	0.09	0.11	0.08
IL-1β	0.49^*^	0.65^*^	0.05	0.27	0.27	−0.03
IL-10	0.68^*^	0.68^*^	0.16	0.32	0.39	−0.35
IL-17A	0.63^*^	0.47^*^	0.20	0.14	0.31	−0.17
Fractalkine	0.36	0.46^*^	0.02	0.24	0.21	−0.12
Granzyme B	0.60^*^	0.63^*^	−0.02	0.15	0.17	0.27

Values are Spearman’s rho; *significant correlation (*p* < 0.05)

*Hypertrophy* quantitative grayscale area (×10^3^ pixel counts), *vascularity* quantitative power Doppler area (×10^3^ pixel counts), *echogenicity* quantitative gray value of grayscale area, *IL* interleukin, *VEG*F vascular endothelial growth factor, *TNF* tumor necrosis factor

In the treated patients with RA, the associations between synovial hypertrophy and any cytokines or growth factors were diminished, although synovial vascularity remained positively correlated with IL-6 and VEGF (*p* < 0.05); synovial echogenicity was also significant and inversely correlated with IL-6 and VEGF (*p* < 0.05). To validate this result of echogenicity, we converted echogenicity to calibrated optical density, and these also significantly correlated positively with SF IL-6 and VEGF (IL-6, rho = 0.47, *p* = 0.02; VEGF, rho = 0.55, *p* < 0.01. summarized in Additional file 3: Figure S2).

### Association between synovial echogenicity and joint inflammation

**Fig. 1:**
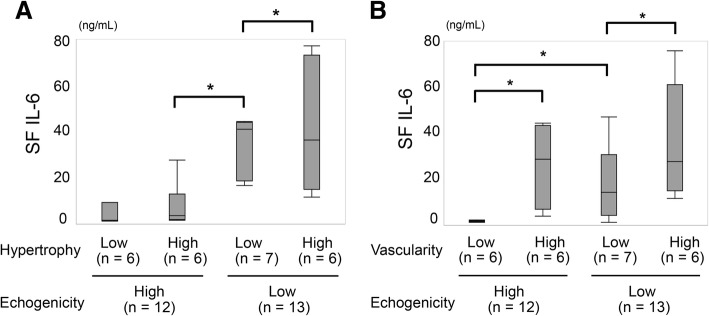
The association between synovial fluid (SF) IL-6 and quantitative findings of ultrasonography in treated patients with rheumatoid arthritis (RA). **a** SF IL-6 is higher in patients with RA and low synovial echogenicity, regardless of their synovial hypertrophy. **b** There was no statistically significant difference in SF IL-6 levels between patients with hyperechogenic synovium with high vascularity and hypoechogenic synovium with low vascularity. GS score, quantitative grayscale area; PD score, quantitative power Doppler area. *Significant correlation (*p* < 0.05)

**Fig. 2:**
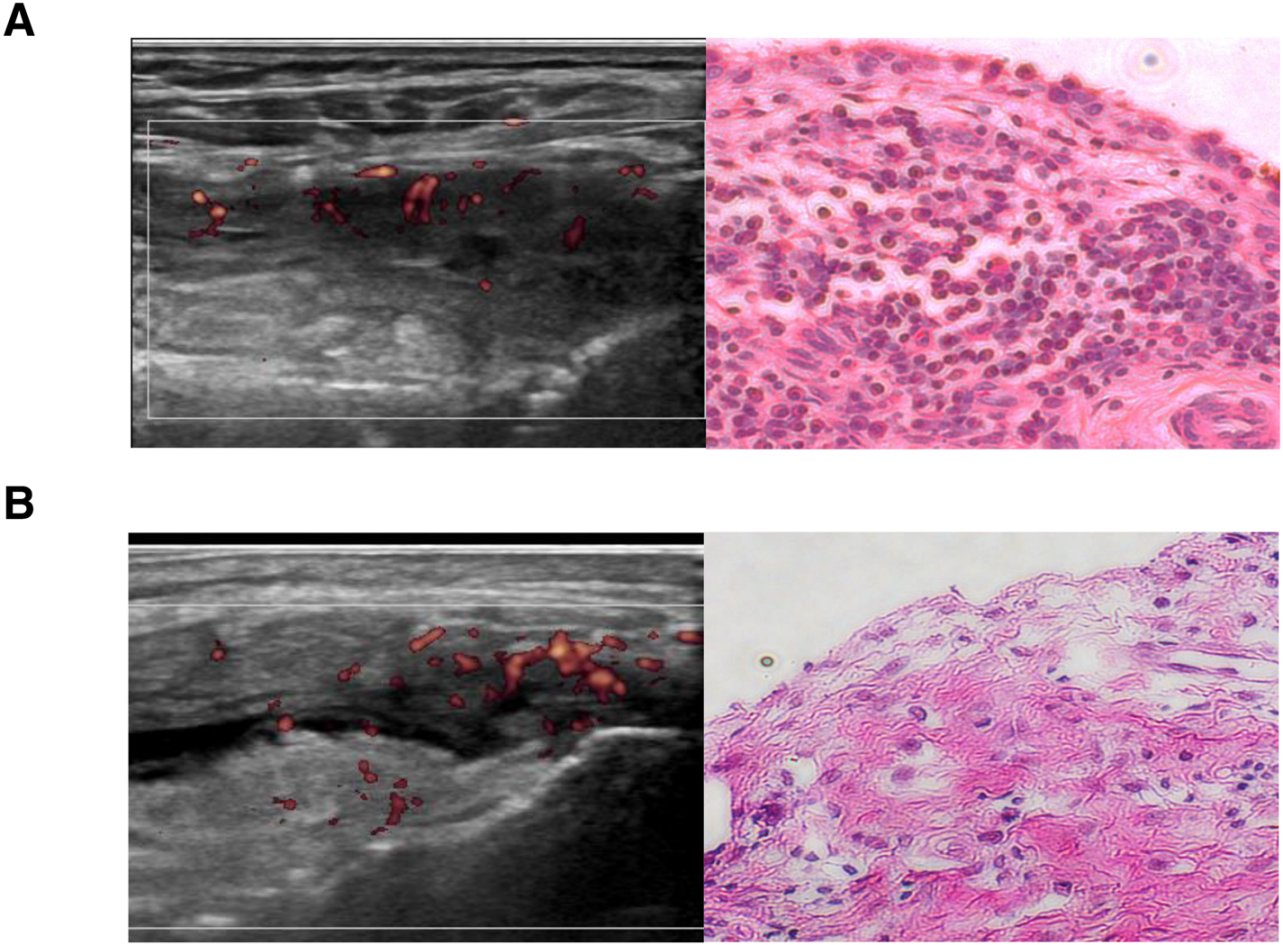
Rheumatoid arthritis (RA) synovial tissue histopathologic appearances directly correlate with the echogenicity of the synovium detected by ultrasonography. **a** Low synovial echogenicity and corresponding histopathologic findings. **b** High synovial echogenicity and histopathologic appearances

We divided the treated patients with RA into two groups based on the median synovial echogenicity and then divided each group into two subgroups using the median pixel counts of synovial hypertrophy or vascularity creating four subgroups to compare SF IL-6 levels, which reveal local inflammatory activity. This analysis showed that SF IL-6 levels were higher in patients with RA with low synovial echogenicity, independent of its hypertrophy, as shown in Fig. 1a. We also confirmed that joint inflammation persisted in patients with low synovial echogenicity, even when synovial vascularity was low (Fig. 1b). Histopathological analysis confirmed that hypoechoic synovial tissue correlated with infiltration of lymphocytes and hypervascularity. Synovium with high echogenicity reflected substantial fibrotic tissue replacement with low-grade mononuclear cell infiltration (Fig. 2).

We found that echogenicity was significantly lower in untreated patients with RA (*p* < 0.01, Additional file 4: Figure S3A). Univariate linear regression analysis showed that synovial echogenicity was associated with RA disease duration in all patients (rho = 0.45, *p* < 0.01, Additional file 4: Figure S3B). Multivariate linear regression analysis using untreated/treated, disease duration, and DAS28-ESR as an independent variable with consideration of multicollinearity revealed that receiving RA treatment was independently associated with synovial echogenicity (*p* < 0.01, Table 3).

**Table 3:** Multivariate linear regression model for synovial echogenicity

	*t* value	Std B	95% CI for B		*p* value
			Lower	Upper	
Treated patients with RA	5.83	0.80	3.83	7.91	< 0.01*
Disease duration	−0.92	−0.12	−0.49	0.19	0.36
DAS28-ESR	1.05	−0.09	− 0.64	2.02	0.30

*Std B* standardized β, *CI* confidence interval, *DAS28* disease activity score based on 28

Joints

*Significant value (*p* < 0.05)

We have checked patient’s body mass index (BMI) and amount of synovial fluid and directly measured the thickness of the subcutaneous lesions and supra-patellar pouch. We identified that those parameters were not significantly associated with echogenicity (summarized in Additional file 5: Table S2). We obtained post SF-aspiration images in 14 patients. We identified that the echogenicity of synovial tissue did not differ significantly before and after SF aspiration. (*p* < 0.26).

## Discussion

This study demonstrated the pathophysiological meanings of US findings including grayscale assessment of synovial hypertrophy, PD indications of vascularity, and synovial echogenicity in patients with active RA. Our data showed that PD vascularity correlated with synovial fluid inflammatory cytokines and growth factors with higher coefficient values in both untreated and treated patients with RA. This supports the results of previous clinical studies of US, which found that power Doppler is an important modality to reflect joint inflammation [30–32]. PD vascularity also significantly correlated with lymphangiogenic factors, including VEGF and fractalkine. This result is consistent with the recent study of Kelly et al. that showed an association between PD signal and histopathological synovial vascularity and angiogenic gene expression [14]. VEGF plays an important role in the pathogenesis of RA synovitis and positively correlates with disease activity and development of radiographic damage [33]. Fractalkine, an endothelial membrane-bound chemokine, is also highly expressed in the synovium in RA and has been investigated as a possible biological drug target [34]. From the above, we suggest that PD visualizes not only vascular abnormality in inflamed joints but also it can visualize local lymphangiogenesis in the synovium in RA.

To our knowledge, this is the first study to demonstrate that presence of synovial hypoechogenicity is directly linked to active inflammation, rather than hypertrophy itself, especially in treated patients with RA. We also observed that RA treatment affected synovial echogenicity and these data may explain why older studies did not show the association between synovial histopathology and US findings [17–19]. Based on the results of our study, hypoechogenic synovial hypertrophy is as good an indicator of local inflammation as PD signal in treated patients with RA. It can be useful to assess treatment response and to predict the likelihood of local exacerbation after cessation or reduction of treatment in patients with RA. Echogenicity can be influenced by some anatomical factors including subcutaneous thickness or amount of synovial fluid. We included patients with RA who had almost standard BMI and the changes in echogenicity were more dynamic than those in skin thickness and synovial fluid, thus the influence of those variables on synovial echogenicity was not so big in this study. On another front, we also showed that the GS of synovial hypertrophy is associated with SF inflammatory cytokines in untreated patients with RA. These data can be applied to evaluate large joints such as the knee, hip, and shoulder, which were difficult to assess by physical examination and PD signals in newly diagnosed patients with RA.

Limitations of this study include the fact that we examined only nine cytokines and growth factors that are well-known to be critical in RA. Second, histopathological analysis was performed only in two patients. That is because the safety and tolerability of US-guided synovial biopsy has not been established in our country. We are now confirming the safety of this procedure in another prospective clinical study in Japan and we would like to conduct a more intensive study in the future. Third, although we examined cytokine levels in synovial fluid, we did not evaluate cytokine expression in synovial tissue. Fourth, our study did not identify the clinical prognosis of treated patients with RA with synovial hypoechogenicity, primarily due to the wide variety of their treatments. This should be studied in the future.

## Conclusion

We demonstrated the pathophysiological meaning of US synovial hypertrophy, vascularity, and echogenicity and which of them are linked to synovial fluid cytokines and inflammatory processes, respectively. PD signals represent active synovitis in any RA clinical situation. Grayscale synovial hypertrophy was not indicative of inflammation but synovial hypoechogenicity is an alternative indicator of persistent joint inflammation, especially in treated patients with RA.

## Additional files

Additional file 1: **Table S1.** Patient (*n* = 44) characteristics, ultrasonography and synovial fluid cytokines. (DOCX 46 kb)

Additional file 2: **Figure S1.** The relationships of quantitative and semi-quantitative ultrasonography findings. (A) Quantitative synovial hypertrophy and GS score are significantly correlated. (B) Quantitative PD vascularity and PD score are also correlated. ^*^Significant value. (TIF 374 kb)

Additional file 3: **Figure S2.** The relationships between SF IL-6 and VEGF levels and synovial brightness in under-treated patients with RA. (A) SF IL-6 and VEGF are inversely correlated with synovial echogenicity. (B) SF IL-6 and VEGF also correlated with optical density converted from echogenicity. (TIF 494 kb)

Additional file 4: **Figure S3.** Factors correlating with synovial brightness detected by ultrasonography. (A) Knee synovial echogenicity in treated patients with RA was significantly higher than that in untreated patients (*p* < 0.01). (B) Synovial echogenicity significantly correlated with RA disease duration (rho = 0.45, *p* = 0.02). ^*^Significant value. (TIF 397 kb)

Additional file 5: **Table S2.** Correlation between synovial echogenicity and possible influencers. (DOCX 44 kb)

## Abbreviations

ACPA: Anti-citrullinated protein antibody
Anti-CCP: Anti-cyclic citrullinated peptide antibody
ACR: American College of Rheumatology
BMI: Body mass index
brightness: Quantitative measurement of pixel counts and gray values
CRP: C-reactive protein
DAS28-ESR: Disease activity score based on 28 joints/erythrocyte sedimentation rate
DMARDs: Disease-modifying anti-rheumatic drugs
Echogenicity: Quantitative gray value of synovial area
ESR: Erythrocyte sedimentation rate
EULAR: European League Against Rheumatism
GS score: Semi-quantitative scoring of synovial hypertrophy
GS: Grayscale
Hypertrophy: Quantitative synovial grayscale area (× 10^3^ pixels)
ICC: Intraclass correlation coefficient
IL: Interleukin
IQR: Interquartile range
MMP-3: Matrix metalloproteinase-3
PD score: Semi-quantitative scoring of synovial vascularity
PD: Power Doppler
RA: Rheumatoid arthritis
RF: Rheumatoid factor
ROIs: Regions of interest
r_s_: Approximate Spearman’s rho
SD: Standard deviation
SF: Synovial fluid
TNF-α: Tumor necrosis factor alpha
US: Ultrasonography
Vascularity: Quantitative synovial power-Doppler area (× 10^3^ pixels)
VEGF: Vascular endothelial growth factor

## References

[1] Wakefield RJ, Balint PV, Szkudlarek M, Filippucci E, Backhaus M, D'Agostino MA (2005). Musculoskeletal ultrasound including definitions for ultrasonographic pathology. J Rheumatol.

[2] Conaghan PG, O'Connor P, McGonagle D, Astin P, Wakefield RJ, Gibbon WW (2003). Elucidation of the relationship between synovitis and bone damage: a randomized magnetic resonance imaging study of individual joints in patients with early rheumatoid arthritis. Arthritis Rheum.

[3] Strunk J, Lange U (2004). Three–dimensional power Doppler sonographic visualization of synovial angiogenesis in rheumatoid arthritis. J Rheumatol.

[4] Ju JH, Yoon CH, Kim HY, Park SH (2007). Clinical images: visualization of the inner synovial surface with three-dimensional ultrasonography. Arthritis Rheum.

[5] Ikeda K, Nakagomi D, Sanayama Y, Yamagata M, Okubo A, Iwamoto T (2013). Correlation of radiographic progression with the cumulative activity of synovitis estimated by power Doppler ultrasound in rheumatoid arthritis: difference between patients treated with methotrexate and those treated with biological agents. J Rheumatol.

[6] Iwamoto T, Ikeda K, Hosokawa J, Yamagata M, Tanaka S, Norimoto A (2014). Prediction of relapse after discontinuation of biologic agents by ultrasonographic assessment in patients with rheumatoid arthritis in clinical remission: high predictive values of total gray-scale and power Doppler scores that represent residual synovial inflammation before discontinuation. Arthritis Care Res.

[7] Backhous M (2009). Ultrasound and structural changes in inflammatory arthritis: synovitis and tenosynovitis. Ann N Y Acad Sci.

[8] Saleem B, Keen H, Goeb V, Parmar R, Nizam S, Hensor EM (2010). Patients with RA in remission on TNF blockers: when and in whom can TNF blocker therapy be stopped?. Ann Rheum Dis.

[9] Humby F, Kelly S, Hands R, Rocher V, DiCicco M, Ng N (2015). Use of ultrasound–guided small joint biopsy to evaluate the histopathologic response to rheumatoid arthritis therapy: recommendations for application to clinical trials. Arthritis Rheum.

[10] Walther M, Harms H, Krenn V, Radke S, Kirschner S, Gohlke F (2002). Synovial tissue of the hip at power Doppler US: correlation between vascularity and power Doppler US signal. Radiology.

[11] Koski JM, Saarakkala S, Helle M, Hakulinen U, Heikkinen JO, Hermunen H (2006). Power Doppler ultrasonography and synovitis: correlating ultrasound imaging with histopathological findings and evaluating the performance of ultrasound equipments. Ann Rheum Dis.

[12] Pitzalis C, Kelly S, Humby F (2013). New learnings on the pathophysiology of RA from synovial biopsies. Curr Opin Rheumatol.

[13] Walther M, Harms H, Krenn V, Radke S, Faehndrich TP, Gohlke F (2001). Correlation of power Doppler sonography with vascularity of the synovial tissue of the knee joint in patients with osteoarthritis and rheumatoid arthritis. Arthritis Rheum.

[14] Kelly S, Bombardieri M, Humby F, Ng N, Marrelli A, Riahi S (2015). Angiogenic gene expression and vascular density are reflected in ultrasonographic features of synovitis in early rheumatoid arthritis: an observational study. Arthritis Res Ther.

[15] Fukae J, Tanimura K, Atsumi T, Koike T (2014). Sonographic synovial vascularity of synovitis in rheumatoid arthritis. Rheumatology (Oxford).

[16] Andersen M, Ellegaard K, Hebsgaard JB, Christensen R, Torp-Pedersen S, Kvist PH (2014). Ultrasound colour Doppler is associated with synovial pathology in biopsies from hand joints in rheumatoid arthritis patients: a cross-sectional study. Ann Rheum Dis.

[17] Bugatti S, Manzo A, Bombardieri M, Vitolo B, Humby F, Kelly S (2011). Synovial tissue heterogeneity and peripheral blood biomarkers. Curr Rheumatol Rep.

[18] Takase K, Ohno S, Takeno M, Hama M, Kirino Y, Ihata A (2012). Simultaneous evaluation of long-lasting knee synovitis in patients undergoing arthroplasty by power Doppler ultrasonography and contrast-enhanced MRI in comparison with histopathology. Clin Exp Rheumatol.

[19] Caporali R, Smolen JS (2018). Back to the future: forget ultrasound and focus on clinical assessment in rheumatoid arthritis management. Ann Rheum Dis.

[20] Arend WP, Dayer JM (1990). Cytokines and cytokine inhibitors or antagonists in rheumatoid arthritis. Arthritis Rheum.

[21] Kokebie R, Aggarwal R, Lidder S, Hakimiyan AA, Rueger DC, Block JA (2011). The role of synovial fluid markers of catabolism and anabolism in osteoarthritis, rheumatoid arthritis and asymptomatic organ donors. Arthritis Res Ther.

[22] Kubota E, Kubota T, Matsumoto J, Shibata T, Murakami KI (1998). Synovial fluid cytokines and proteinases as markers of temporomandibular joint disease. J Oral Maxillofac Surg.

[23] Karsdal MA, Woodworth T, Henriksen K, Maksymowych WP, Genant H, Vergnaud P (2011). Biochemical markers of ongoing joint damage in rheumatoid arthritis––current and future applications, limitations and opportunities. Arthritis Res Ther.

[24] van den Ham HJ, de Jager W, Bijlsma JW, Prakken BJ, de Boer RJ (2009). Differential cytokine profiles in juvenile idiopathic arthritis subtypes revealed by cluster analysis. Rheumatology (Oxford).

[25] Arnett FC, Edworthy SM, Bloch DA, McShane DJ, Fries JF, Cooper NS (1988). The American Rheumatism Association 1987 Revised criteria for the classification of rheumatoid arthritis. Arthritis Rheum.

[26] Aletaha D, Neogi T, Silman AJ, Funovits J, Felson DT, Bingham CO (2010). 2010 Rheumatoid arthritis classification criteria: an American College of Rheumatology/European League against Rheumatism Collaborative Initiative. Ann Rheum Dis.

[27] Szkudlarek M, Court-Payen M, Strandberg C, Klarlund M, Klausen T, Ostergaard M (2001). Power Doppler ultrasonography for assessment of synovitis in the metacarpophalangeal joints of patients with rheumatoid arthritis: a comparison with dynamic magnetic resonance imaging. Arthritis Rheum.

[28] Koski JM, Saarakkala S, Helle M, Hakulinen U, Heikkinen JO, Hermunen H (2006). Assessing the intra– and inter–reader reliability of dynamic ultrasound images in power Doppler ultrasonography. Ann Rheum Dis.

[29] Kelly S, Humby F, Filer A, Ng N, Di Cicco M, Hands RE (2015). Ultrasound-guided synovial biopsy: a safe, well-tolerated and reliable technique for obtaining high–quality synovial tissue from both large and small joints in early arthritis patients. Ann Rheum Dis.

[30] Ohrndorf S, Backhaus M (2013). Advances in sonographic scoring of rheumatoid arthritis. Ann Rheum Dis.

[31] Naredo E, Bonilla G, Gamero F, Uson J, Carmona L, Laffon A (2005). Assessment of inflammatory activity in rheumatoid arthritis: a comparative study of clinical evaluation with grey scale and power Doppler ultrasonography. Ann Rheum Dis.

[32] Carotti M, Salaffi F, Manganelli P, Salera D, Simonetti B, Grassi W (2002). Power Doppler sonography in the assessment of synovial tissue of the knee joint in rheumatoid arthritis: a preliminary experience. Ann Rheum Dis.

[33] Taylor PC (2002). Arthritis Res.

[34] Tanaka Y, Takeuchi T, Umehara H, Nanki T, Yasuda N, Tago F (2018). Safety, pharmacokinetics, and efficacy of E6011, an antifractalkine monoclonal antibody, in a first-inpatient phase 1/2 study on rheumatoid arthritis. Mod Rheumatol.

