# Supplementary material for: Significant association between joint ultrasonographic parameters and synovial inflammatory factors in rheumatoid arthritis
Source: Arthritis Res Ther. 2019 Jan 10;21:14. doi: 10.1186/s13075-018-1802-x (PMC6327469; doi:10.1186/s13075-018-1802-x)
Supplement: Supplementary file 1 — Table S1. Patient (n = 44) characteristics, ultrasonography and synovial fluid cytokines. (DOCX 46 kb) [file 13075_2018_1802_MOESM1_ESM.docx]

Table S1 Patient (n = 44) characteristics, ultrasonography and synovial fluid cytokines

| **Characteristics (n=44)** |  | |
| --- | --- | --- |
|  |  | |
| ***Demographics*** |  |  |
| Female n (%) | 37 | (84) |
| Age (year, range) | 64 | (54–72) |
| BMI | 23.0 ± 3.7 |  |
| RF positivity n (%) | 33 | (75) |
| ACPA positivity n (%) | 34 | (77) |
| Treated patients n (%) | 25 | (56) |
| Disease duration (year) | 1.5 | (0.25–5) |
| DAS28-ESR | 5.2 ± 1.4 |  |
| CRP (mg/dL) | 3.2 ± 2.9 |  |
| ESR (mm/h) | 74.8 ± 36.7 |  |
| MMP-3 (ng/mL) | 405.1 ± 295.0 |  |
| Synovial fluid cell count /μL | 6883 ± 5607 |  |
| ***Ultrasonography findings*** |  |  |
| GS score | 2.7 | (2.3–2.8) |
| PD score | 2.0 | (1.5–2.3) |
| Hypertrophy (×10^3^ pixel counts) | 137.3 ± 45.7 |  |
| Vascularity (×10^3^ pixel counts) | 18.3 ± 14.4 |  |
| Echogenicity | 46.6 ± 7.6 |  |
| ***Synovial fluid Cytokines*** |  |  |
| IL-6 (ng/mL) | 24.0 ± 24.7 |  |
| VEGF (pg/mL) | 666.8 ± 531.6 |  |
| TNF-α (pg/mL) | 140.8 ± 411.5 |  |
| IL-8 (pg/mL) | 1724.8 ± 1805.9 |  |
| IL-1β (pg/mL) | 34.9 ± 34.0 |  |
| IL-10 (pg/mL) | 32.0 ± 20.2 |  |
| IL-17A (pg/mL) | 44.0 ± 62.3 |  |
| Granzyme B (pg/mL) | 83.2 ± 87.0 |  |
| Fractalkine (pg/mL) | 149.6 ± 234.0 |  |

Values are mean ± SD or median (IQR) unless otherwise specified.

BMI, body mass index; RF, rheumatoid factor; ACPA, anti-citrullinated protein antibody; DAS28, Disease Activity Score based on 28 joints; CRP, C-reactive protein; ESR, erythrocyte sedimentation rate; MMP-3, matrix metalloproteinase-3; GS, grey-scale; PD, power-Doppler; Hypertrophy, quantitative grayscale area (×10^3^ pixel counts); Vascularity, quantitative power-Doppler area (×10^3^ pixel counts); Echogenicity, quantitative gray value of grayscale area; IL, interleukin; VEGF, vascular endothelial growth factor; TNF, tumor necrosis factor
