# Supplementary figures and images for: Significant association between joint ultrasonographic parameters and synovial inflammatory factors in rheumatoid arthritis
Source: Arthritis Res Ther. 2019 Jan 10;21:14. doi: 10.1186/s13075-018-1802-x (PMC6327469; doi:10.1186/s13075-018-1802-x)

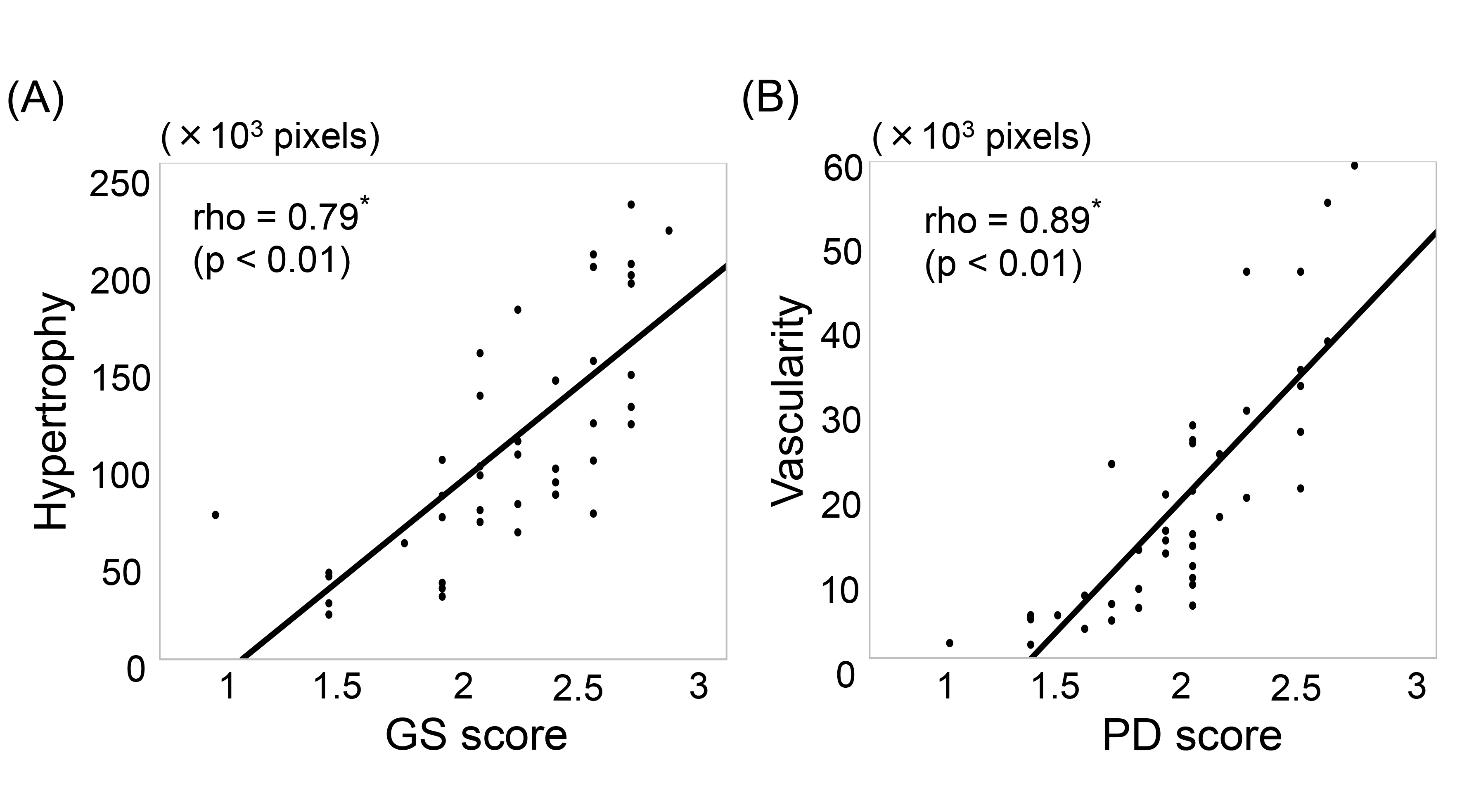

Supplement: Supplementary file 2 — Figure S1. The relationships of quantitative and semi-quantitative ultrasonography findings. (A) Quantitative synovial hypertrophy and GS score are significantly correlated. (B) Quantitative PD vascularity and PD score are also correlated. *Significant value. (TIF 374 kb) [file 13075_2018_1802_MOESM2_ESM.tif]

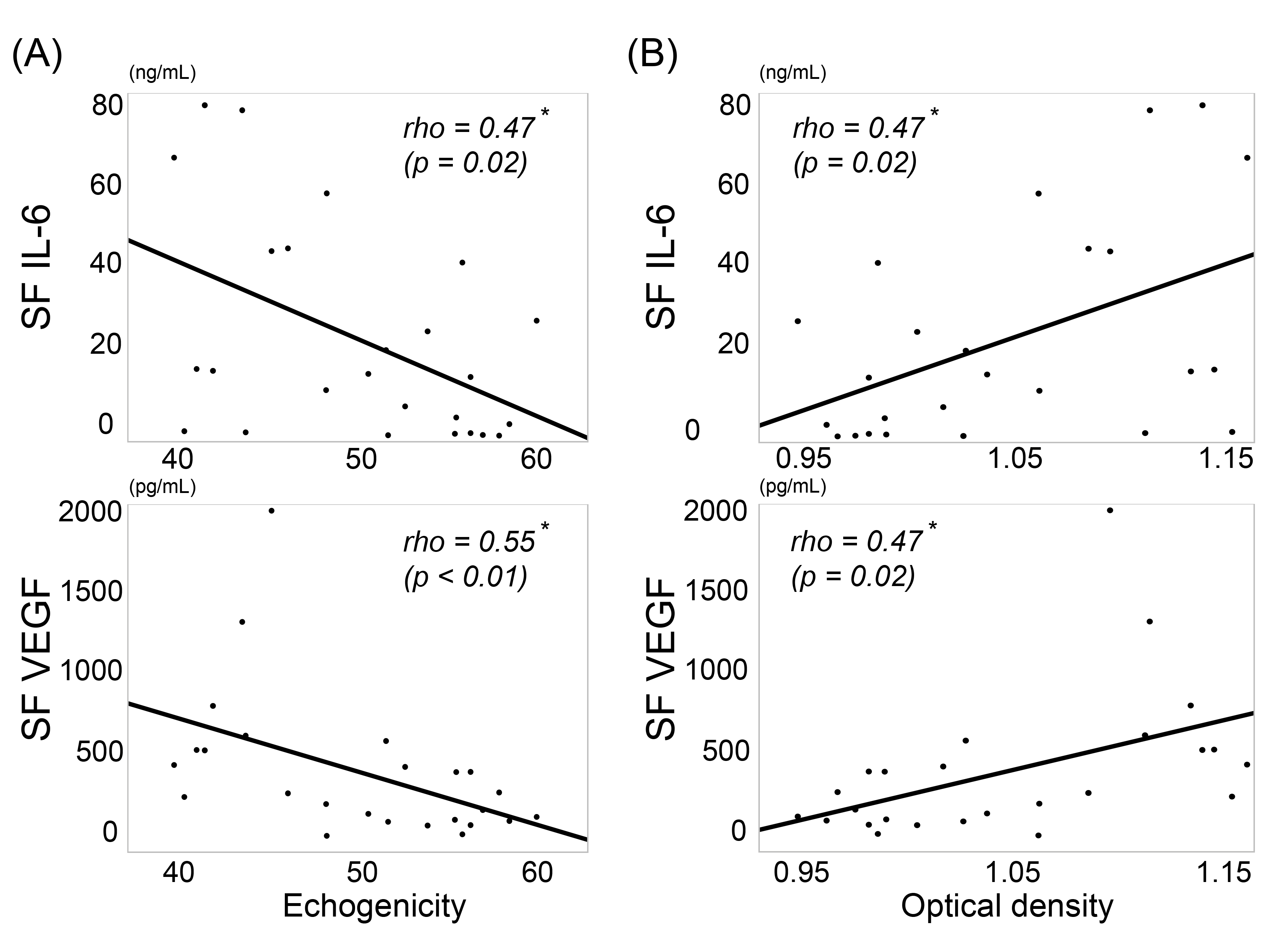

Supplement: Supplementary file 3 — Figure S2. The relationships between SF IL-6 and VEGF levels and synovial brightness in under-treated patients with RA. (A) SF IL-6 and VEGF are inversely correlated with synovial echogenicity. (B) SF IL-6 and VEGF also correlated with optical density converted from echogenicity. (TIF 494 kb) [file 13075_2018_1802_MOESM3_ESM.tif]

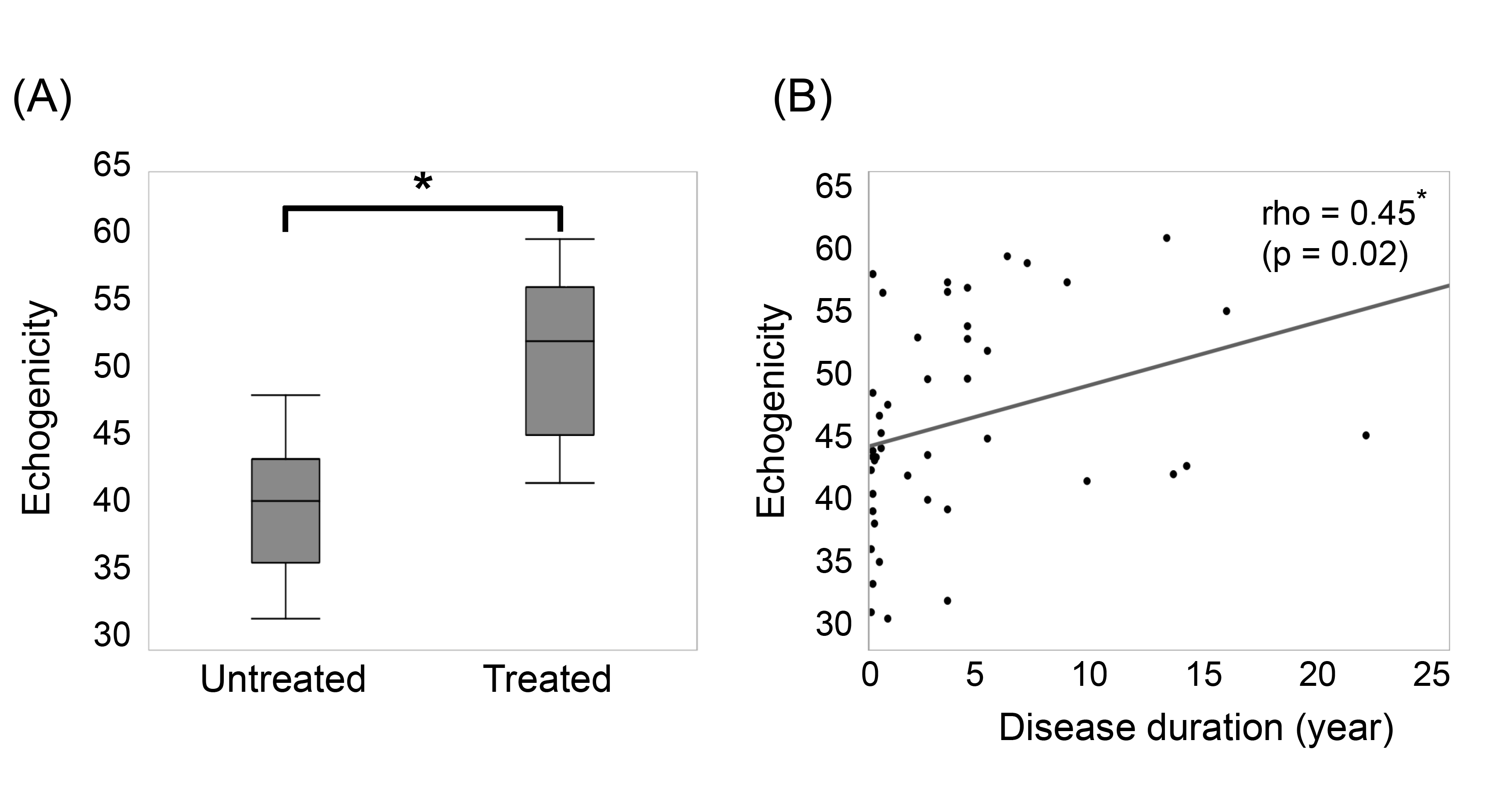

Supplement: Supplementary file 4 — Figure S3. Factors correlating with synovial brightness detected by ultrasonography. (A) Knee synovial echogenicity in treated patients with RA was significantly higher than that in untreated patients (p < 0.01). (B) Synovial echogenicity significantly correlated with RA disease duration (rho = 0.45, p = 0.02). *Significant value. (TIF 397 kb) [file 13075_2018_1802_MOESM4_ESM.tif]
