# Supplementary material for: Significant association between joint ultrasonographic parameters and synovial inflammatory factors in rheumatoid arthritis
Source: Arthritis Res Ther. 2019 Jan 10;21:14. doi: 10.1186/s13075-018-1802-x (PMC6327469; doi:10.1186/s13075-018-1802-x)
Supplement: Supplementary file 5 — Table S2. Correlation between synovial echogenicity and possible influencers. (DOCX 44 kb) [file 13075_2018_1802_MOESM5_ESM.docx]

Table S2. Correlation between synovial echogenicity and possible influencers

| **Parameters** | **Spearman’s rho** | **p value** |
| --- | --- | --- |
| BMI | 0.08 | 0.55 |
| Subcutaneous thickening | 0.05 | 0.58 |
| Amount of synovial fluid | -0.14 | 0.32 |
| Thickness of Supra-patellar pouch | -0.08 | 0.55 |

BMI, body mass index
